# Misspecification of at‐risk periods and distributional assumptions in estimating COPD exacerbation rates: The resultant bias in treatment effect estimation

**DOI:** 10.1002/pst.1798

**Published:** 2016-12-14

**Authors:** M. Law, M.J. Sweeting, G.C. Donaldson, J.A. Wedzicha

**Affiliations:** ^1^ MRC Biostatistics UnitCambridgeUK; ^2^ Department of Public Health and Primary CareUniversity of CambridgeCambridgeUK; ^3^ National Heart and Lung InstituteImperial College LondonCambridgeUK

**Keywords:** chronic obstructive pulmonary disease, exacerbation rate, negative binomial, recurrent events

## Abstract

In trials comparing the rate of chronic obstructive pulmonary disease exacerbation between treatment arms, the rate is typically calculated on the basis of the whole of each patient's follow‐up period. However, the true time a patient is at risk should exclude periods in which an exacerbation episode is occurring, because a patient cannot be at risk of another exacerbation episode until recovered. We used data from two chronic obstructive pulmonary disease randomized controlled trials and compared treatment effect estimates and confidence intervals when using two different definitions of the at‐risk period. Using a simulation study we examined the bias in the estimated treatment effect and the coverage of the confidence interval, using these two definitions of the at‐risk period. We investigated how the sample size required for a given power changes on the basis of the definition of at‐risk period used. Our results showed that treatment efficacy is underestimated when the at‐risk period does not take account of exacerbation duration, and the power to detect a statistically significant result is slightly diminished. Correspondingly, using the correct at‐risk period, some modest savings in required sample size can be achieved. Using the proposed at‐risk period that excludes recovery times requires formal definitions of the beginning and end of an exacerbation episode, and we recommend these be always predefined in a trial protocol.

## 1 INTRODUCTION

Chronic obstructive pulmonary disease (COPD) is a common chronic respiratory disorder. It has a growing societal effect because of high morbidity and mortality.^1^ In the European Union the total direct health care costs for COPD exceeds 23 billion Euros.^2^ Exacerbations are the major cost drivers in COPD, and therefore, reduction in their frequency is an important goal in the therapeutic management of the disease. In the development of new drugs, late phase clinical trials are significant, with the estimated cost in 2014 of bringing a prescription medicine to market in excess of 2.5 billion dollars according to a study from the Turfs Center for the Study of Drug Development (http://csdd.tufts.edu/files/uploads/cost_study_backgrounder.pdf). Thus, any reduction in the required sample size necessary to provide sufficient statistical power will be important.

For chronic conditions that have episodic health effects, such as COPD, which may be characterized by periodic exacerbation of symptoms, a common aim is to estimate the ratio of the rate at which exacerbation episodes occur in 2 treatment groups. In this paper we define a COPD exacerbation episode to consist of a primary exacerbation onset time, defined for example by symptomatic criteria hitting a threshold, followed by a recovery period in which further fluctuations in symptoms and lung function may occur before the patient has deemed to have fully recovered. The length of the recovery period has previously been defined using a 3‐day moving average of a symptom score^3^ or peak flow^4^ or a combination of symptom‐free days and peak flow^5^ returning to preexacerbation levels, or time until a number of symptom‐free days have been reached.^6^ However, it has also been acknowledged that some recovery definitions can be problematic, with a number of episodes not returning to baseline levels,^7^ and hence, a maximum duration cut‐off or degree of expert judgment is sometimes required. Within an episode it is entirely possible for symptom or lung function fluctuations to go beyond the threshold used to define the initial exacerbation event (ie, a worsening of symptoms), but these are generally not considered to indicate a new event. It is not the purpose of this paper to make recommendations regarding the definition of exacerbation recovery; the statistical methods that we present are valid regardless of the definition used, and further, for any episodic recurrent event data.

An individual's exacerbation rate is often derived by dividing the number of episodes they experience by their time under follow‐up to obtain their estimated exacerbation rate per year.^8^ The distribution of these rates can then be compared between the 2 treatment groups using a nonparametric test, such as the Wilcoxon rank‐sum test.^9^, ^10^ Alternatively, a model‐based analysis is often recommended, whereby the number of exacerbation episodes experienced by a patient is modeled using a negative binomial distribution, while accounting for their at‐risk period.^9^, ^11^ The at‐risk period is usually specified as their total follow‐up time.^8^ However, for these approaches, an individual's at‐risk period is misspecified as it is not typical for a patient experiencing an exacerbation episode to concurrently suffer the onset of another episode. A patient experiencing an exacerbation episode is thus not at risk of another until recovered.^12^

Data such as this—recurrent events with associated duration, during which a subsequent event is not possible—have been described by Cook and Lawless.^13^ This definition of at‐risk period is not typically reflected in the analysis of COPD exacerbation rates. Estimating an exacerbation rate using the number of days each patient is truly at risk would result in a higher rate of exacerbation than is usually estimated and a more accurate estimation of the true treatment effect. In this paper we shall henceforth refer to the typical at‐risk measure (total length of follow‐up in the trial) as the always at risk (AAR) definition and the proposed at‐risk measure (duration of follow‐up spent not recovering) as the excluding recovery time (ERT) definition.

Previous authors have shown that there are a number of ways in which COPD trial results may be suboptimal through conducting incorrect statistical analyses of exacerbation rates.^8^, ^9^, ^11^, ^14^ These include analyses that do not weight each patient's estimated exacerbation rate by their length of follow‐up or models that do not properly account for heterogeneity. Such variation, if it is evident, should be properly modeled. Noting that a standard Poisson regression analysis would lead to overprecise estimates, Keene et al^9^ suggested the use of a negative binomial regression model to take account of overdispersion and illustrate the effect of fitting such a model to two COPD randomized trials.

In this paper, we used data from two previous trials: one trial investigated how prescription of the macrolide erythromycin could reduce COPD exacerbation rate,^6^ while the other examined the effect of long‐term antibiotics on airway bacteria and rates of exacerbation.^15^ We investigate how robust the trial results are to at‐risk period. We quantify, using simulation, the difference in the estimated (mean) exacerbation rates between using the AAR and ERT at‐risk definitions under a number of scenarios, whereby the true rates of exacerbation and recovery are varied between two treatment groups. We examine the reduction in sample size (and equivalent gains in power) that can be made by using the more appropriate ERT at‐risk definition over AAR. All results are discussed in the context of future clinical trial practice.

## 2 METHODS

### 2.1 Clinical interpretation of at‐risk periods

Redefining the at‐risk period requires clarifying the interpretation of the resulting exacerbation rate. Under the typical definition of at‐risk period, AAR, a patient is deemed to be at risk of an exacerbation at all times during follow‐up. Exacerbation rate is generally stated per person‐year and is interpreted as the mean number of exacerbations a patient experienced in one year of follow‐up. Under our updated definition of at‐risk period, ERT, a patient is deemed at be at risk of an exacerbation only when that patient is not experiencing an exacerbation—that is, it is not possible for a patient to experience two or more exacerbations simultaneously. This definition differentiates between time spent in exacerbation and not in exacerbation, taking only the latter as the at‐risk period. Thus, the clinical interpretation describes the mean *time between* periods of exacerbation. For example, a patient may experience two exacerbations over a single year of follow‐up, with each exacerbation lasting 1 month. The exacerbation rate under AAR would be two exacerbations per person‐year. Under ERT, the rate would be calculated as 2 exacerbations over 10 months (or 0.833 year) of being at risk, hence a mean of 2/0.833 = 2.401 exacerbations per year of at‐risk time. Using the ERT definition of at‐risk period takes account of this difference, while AAR does not. This definition of at‐risk period for respiratory exacerbation has been used previously by Therneau and Hamilton^12^ and more recently by Donaldson et al.^5^

Exacerbation onset and recovery is generally inferred from daily diary cards completed by the patients, on which their symptoms are recorded. If such records are inaccurate, our proposed at‐risk definition is of less use. However, patients may be “trained” to use the diary cards by administering them in advance of receiving their study drug and examining their entries at regular intervals.^15^ In studies that use electronic handheld devices to record daily symptoms, alerts are typically set up to remind patients to complete data. In such studies, inference may be made using the recorded exacerbation durations, even over longer‐term follow‐up.^5^ Nevertheless, exact exacerbation onset and recovery dates may be difficult to discern in some cases, even when strict definitions are in place. In such cases, expert opinion may be sought (see Discussion section).

### 2.2 Negative binomial regression

An analysis of exacerbations may be subject based or time based^11^: briefly, a subject‐based approach involves obtaining a rate for each patient individually by dividing a patient's number of exacerbations by their length of follow‐up time, and then taking the average (mean or median) across the treatment group; thus, all patients contribute equally to an analysis regardless of follow‐up time. A time‐based approach involves estimating the mean rate in a treatment group directly by summing all patients' exacerbations and dividing by the total follow‐up time; thus, patients with longer follow‐up make a greater contribution to the analysis than patients with shorter follow‐up times. Parametric models are used to estimate a confidence interval and *P* value for the rate ratio. A more thorough explanation has previously been given by Suissa.^8^

One parametric approach to account for unmeasured between‐subject heterogeneity (ie, heterogeneity not explained by patient characteristics) is to use negative binomial regression. Each patient's expected number of events is considered to be a function of the covariates, but also a random (latent) variable, which increases or decreases the patient's expected number of events depending, for example, on whether he or she is a frequent exacerbator or not, or has chronic bacterial colonization in their lungs or not. Let *y*_*i*_ denote the number of exacerbations observed for patient *i* over an at‐risk period *T*_*i*_, where the at‐risk period is either specified as the total length of follow‐up for patient *i* *,* or the length of follow‐up spent not in exacerbation, depending on whether we are respectively estimating the treatment effect using the AAR or ERT definition of at‐risk period. Then let

$$\begin{array}{c} \left(y_{i}|\upsilon_{i}\right)∼\text{Poisson}\left(\theta_{i}\upsilon_{i}\right)\\ \log\left(\theta_{i}\right)=\beta x_{i}+\log\left(T_{i}\right) \end{array}$$

where *θ*_*i*_ is the expected number of exacerbations experienced by patient *i* over at‐risk period *T*_*i*_, ***x***_*i*_ is a vector of covariate values for patient *i*, **β** is the corresponding vector of parameter estimates (which may include an intercept term), and *υ*_*i*_ is the random effect for patient *i*. If these random effects across patients follow a Gamma distribution with unit mean and variance *τ*, then the marginal distribution of *y*_*i*_ follows a negative binomial distribution with mean *θ*_*i*_ and overdispersion parameter *τ*. The exacerbation rate of patient *i* is $\mu_{i}=\frac{\theta_{i}\nu_{i}}{T_{i}}=\exp\left(\beta x_{i}\right)\nu_{i}$. In particular, if **β*x***_*i*_ = *β*_0_ + *β*_1_*Z*_*i*_, where *Z*_*i*_ is the treatment indicator (1 = drug, 0 = placebo), then *β*_0_ is the mean log exacerbation rate in the Placebo group, and *β*_1_ is the log rate‐ratio for drug versus placebo (the treatment effect)*.* Here, the offset term is log(*T*_*i*_) and accounts for differences in follow‐up periods that are caused by dropout and the differing lengths of time patients spend in exacerbation recovery (if using the ERT definition).

It has been suggested that the negative binomial model be used with exacerbation count data whenever there is a possibility of heterogeneity in exacerbation rates.^8^ In some trials^16^, ^17^ overdispersion correction is accounted for after fitting a Poisson regression model. This is an approach discussed by Suissa.^8^ However, this approach merely increases the standard error of the resulting estimates and does not affect the estimate of the treatment effect, which could be underestimated.^9^ The negative binomial model may be compared to approaches that allow a more general variance function, estimated using quasi‐likelihood methods; however, this is outside the remit of this paper.

## 3 APPLICATION TO A MACROLIDE STUDY

Using the methods described in the previous section, we used data from a two‐arm randomized controlled trial^6^ where COPD patients were allocated to receive either the macrolide erythromycin or a placebo in addition to their usual medication, over a 1 year period. To estimate the exacerbation rate ratio the study used a Poisson regression model with log time on treatment as the offset variable and adjusted for baseline covariates of smoking status, number of exacerbations in the previous 12 months, age and disease severity. Characteristics of the study are recorded in Table 1.

**Table 1:** Characteristics, results, and reanalysis of the work of Seemungal et al^6^

		**Erythromycin**	**Placebo**
**N**		53	56
**Exacerbation frequency; median (IQR)**		1.00 (0.00, 2.00)	1.00 (0.0, 3.25)
**Total exacerbations**		81	125
**Exacerbation duration, days; median (IQR)** a		9 (6, 14)	13 (7, 24)
		**Treatment effect (rate ratio (95% CI))**	
**Empirical with Wilcoxon test**	**AAR**	0.61, *P* = 0.02^b^	—
	**ERT**	0.22, *P* = 0.01^b^	—
**Negative binomial regression**	**AAR**	0.61 (0.39, 0.96) *P* = 0.03^c^	—
	**ERT**	0.54 (0.33, 0.89) *P* = 0.02^c^	—

a Duration known for only 68 exacerbations in the erythromycin group, 96 exacerbations in the placebo group.

b P values calculated using Wilcoxon rank‐sum test.

c P values calculated using Wald test.

Abbreviations: AAR, always at risk estimation of at‐risk period; ERT, excluding recovery time estimation of at‐risk period.

There were missing data regarding exacerbation duration: 13 (16%) in the treatment group and 29 (23%) in the placebo group. We have imputed the missing values using single imputation of the mean duration in each arm—18.89 days in the control group and 13.50 days in the treatment group. We accept that the distribution of exacerbation duration is skewed, and one may reasonably choose medians (9.00 on control, 13.00 on treatment) over means. One patient in the control group, who was in the study for 13 days only, experienced an exacerbation of unknown duration. However, as this occurred on the patient's final day of follow‐up, the patient was not assumed to be further at risk after this exacerbation. This analysis uses both the total number of exacerbations experienced and the total number of days spent at risk, calculated by taking the total follow‐up and subtracting exacerbation periods. The analysis of this study data using time in study as the AAR time results in the estimates as shown in Table 1. It is possible to compare the treatment groups without making any distributional assumptions, by taking the mean of the exacerbation rates in each group. This results in an empirical (subject‐based) rate ratio of 0.61 (ie, a 39% reduction in the rate of exacerbation) with a *P* value of *P* = 0.02 using the Wilcoxon rank‐sum test (Table 1). Accounting for the recovery periods using the ERT at‐risk period resulted in the rate ratio of 0.22 (*P* = 0.01), again subject based. Using a negative binomial model more appropriately accounts for between‐subject variability and gives rate ratios of 0.61 for the AAR at‐risk definition and 0.54 for the ERT definition. Both remain statistically significant.

## 4 APPLICATION TO A LONG‐TERM ANTIBIOTIC STUDY

We also analyzed a four‐arm randomized controlled trial^15^ where COPD patients were allocated to receive one of three antibiotics—moxifloxacin, doxycycline, or azithromycin—or a placebo over a 13‐week period. To estimate the rate ratio of exacerbation, the study used a negative binomial regression model with log time on treatment as the offset variable, adjusted for baseline covariates of smoking status, number of exacerbations in the previous 12 months, age, sex, and FEV1 as a percentage of the predicted value. Characteristics of the study are recorded in Table 2.

**Table 2:** Characteristics, results, and reanalysis of the work of Brill et al^15^

	**Moxifloxacin**	**Doxycycline**	**Azithromycin**	**Placebo**
**N**	25	25	25	24
**Exacerbation frequency; median (IQR)**	0.00 (0.00, 1.00)	1.00 (0.00, 2.00)	0.00 (0.00, 1.00)	1.00 (0.00, 1.00)
**Total exacerbations**	21	32	13	15
**Exacerbation duration, days; median (IQR)**	7.00 (4.00, 11.00)	7.00 (4.00, 10.00)	8.00 (6.00, 29.00)	9.00 (6.00, 24.50)
**At‐risk definition**	**Treatment effect (rate ratio) (95% CI)**			
**AAR**	1.38 (0.62, 3.10), *P* = 0.43	2.07 (0.99, 4.35), *P* = 0.05	0.72 (0.30, 1.71), *P* = 0.45	—
**ERT**	1.42 (0.55, 3.64), *P* = 0.47	2.18 (0.90, 5.24), *P* = 0.08	0.76 (0.29, 1.99), *P* = 0.58	—

All *P* values calculated using Wald test.

Abbreviations: AAR, always at risk estimation of at‐risk period; ERT, excluding recovery time estimation of at‐risk period.

## 5 SIMULATION STUDY

A series of 10,000 clinical trials were simulated under varying conditions to assess how the choice of at‐risk period typically affects estimation of exacerbation rate ratios. As the underlying characteristics of the series of trials, including the true exacerbation rates and treatment effect, were known, it was possible to compare the two definitions of follow‐up. We compare the treatment effect estimates under both the AAR and ERT definitions obtained from each set of 10,000 simulated trials with the true known treatment effect to obtain a measure of bias. The bias is defined as the percentage bias—the difference between the estimated and true treatment effects, divided by the true treatment effect. This can be expressed as a percentage by simply multiplying by 100. Note that we define bias here in relationship to the true model, in which exacerbation events are generated under an ERT scenario. We further study the properties of the estimated confidence intervals to assess coverage, which is the proportion of simulations for which the true treatment effect lies within the 95% confidence interval from the simulation, and also examine the power to show a statistically significant difference, given varying true differences.

Treatment effects are estimated under a model that allows between‐subject heterogeneity in the exacerbation rates—i.e., exacerbation counts follow a negative binomial distribution.

### 5.1 Simulation methods

Each series of simulations was comprised of 10,000 realized trials. The default properties of the trials are chosen to be similar to that seen in the COPD macrolide clinical trial^6^ and a trial of Tiotropium by Powrie et al.^4^ In this latter trial, 142 patients were randomized to anticholinergic bronchodilator Tiotropium or placebo, in addition to their regular medication, and followed up for one year. The primary outcome of the study was airway inflammation; however, exacerbation frequency and duration were also recorded. In this series of simulations, each trial is a parallel study with *75* patients per arm randomized into an experimental (*E*) or control (*C*) group. Variable follow‐up times are simulated from a normal distribution, with a mean follow‐up of 365 days and a standard deviation of 30 days. The length of follow‐up is independent of any patient characteristics, and hence, dropout from any trial is completely at random.^18^ In practice, patients with higher rates of exacerbation are more likely to withdraw early.^19^ This is addressed in a further analysis contained within the supplementary material of this paper.

An exacerbation rate for each patient is obtained by multiplying the group‐specific mean exacerbation rate *λ*_*C*_ or *λ*_*E*_ for the control or experimental group, respectively, by *υ*_*i*_ (simulated from a Gamma distribution with unity shape and rate, and hence an overdispersion parameter equals to 1 throughout). The standard deviation of the exacerbation rate is therefore equal to the group‐specific mean exacerbation rate. Recovery from exacerbation occurs at a rate *γ*_*E*_ on experimental treatment and *γ*_*C*_ on control, with no between‐subject variation assumed in the recovery rates other than that explained by treatment.

For each subject, a series of times until next exacerbation and times until recovery (while in exacerbation) are sampled from exponentially distributed random variables using the patient's exacerbation and recovery rates, respectively, until they reach the end of their follow‐up*.* Simulating times to exacerbation in this way is equivalent to simulating exacerbation counts over follow‐up directly from a negative binomial distribution, but with the advantage of allowing recovery periods to be also simulated after each exacerbation occurs. As such, it is then possible to investigate the effects of incorrectly defining time at risk of exacerbation.

A number of scenarios are investigated by changing the parameter values that govern the trial properties (Table 3). The default rate of exacerbation in the control group was chosen to be 1.8 exacerbations per person‐year, while other scenarios allowed the rate to be as low as 1.3 or as high as 2.3 exacerbations per person‐year. In the work of Powrie et al, the mean exacerbation rate on control was 2.46 per person‐year, while in the work of Seemungal et al, the median frequency for one year of follow‐up was 1.00 (Table 1). These studies are thus comparable to the simulation scenarios of exacerbation frequency, sample size (142 in the work of Powrie et al, 109 in the work of Seemungal et al), and follow‐up time (both one year). The treatment effect (rate ratio) was then varied to take values of 0.5 (half the exacerbation rate on treatment compared to control), 0.7, 1.0 (null case), and 1.5 (i.e., exacerbation rate on treatment is 50% higher than control). The recovery rate on control was fixed at *γ*_*C*_ = 36.5 per person‐year, equivalent to a mean exacerbation recovery time of approximately 10 days. The default recovery rate in the experimental group was set equal to that in the control group, and so the mean duration of exacerbations is the same in both arms. However, we also investigated this recovery rate being 50% and 100% greater in the treatment than the control group (i.e., reducing exacerbation duration in the treatment group), while other trial parameters remained fixed at their default values. That is, we investigate the effect of varying the recovery rate. Note that as the absolute recovery rates increase, the results of the AAR and ERT analyses converge in the limit, i.e., when exacerbation duration is zero days.

**Table 3:** Trial design parameters used in the simulation study

**Parameter**	**Value(s) Taken**
Mean exacerbation rate in control group, per‐person year, *λ*_*C*_	1.3, 1.8^a^, 2.3
**Treatment effect, rate ratio *φ***	0.5, 0.7^a^, 1.0, 1.5
**Overdispersion parameter τ**	1.0
Recovery rate in control group, per‐person year, *γ*_*C*_	36.5
Recovery rate ratio, *γ*_*E*_/*γ*_*C*_	1.0^a^, 1.5, 2.0

a It denotes default values.

For each series of simulations, the overall treatment effect was estimated using both the typical measure of follow‐up, AAR, and the more appropriate measure, ERT, under a negative binomial regression model. The percentage bias of the treatment effect (rate ratio) was recorded, together with the coverage of the 95% confidence intervals and the probability of rejecting the null hypothesis (power). When data are simulated in the scenario with no difference between the mean control and experimental exacerbation rates, the probability of rejecting the null hypothesis represents the probability of making a type I error—i.e., of incorrectly favoring a non‐superior treatment. We summarize how the methods affect the probability of type I error.

### 5.2 Simulation results

#### 5.2.1 Bias and coverage

**Figure 1:**
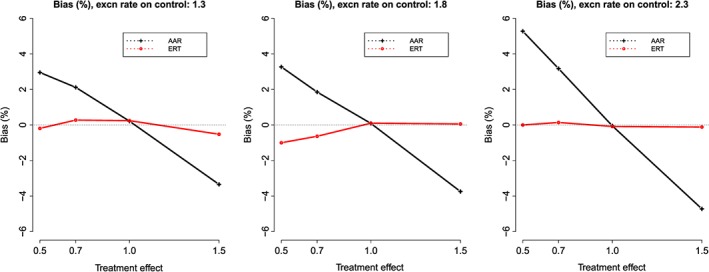
Percentage bias in treatment effect estimate under both AAR and ERT. Results are shown for varying true treatment effect, and varying exacerbation rate in the control group

Figure 1 shows bias for AAR and ERT definitions of at‐risk period, with a recovery rate ratio of 1 (ie a recovery rate of 36.5 per person‐year on both arms). The use of AAR at‐risk weights produced biased estimates of the treatment effect in the range −4.5% to +5.4% of the true effect. The degree of bias increased as the treatment effect increased, and as exacerbation rate increased. The direction of bias was always toward the null, thus underestimating the difference between the treatment groups.

**Figure 2:**
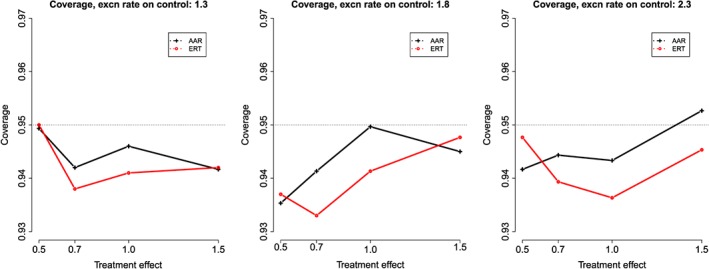
Coverage probability of treatment effect confidence interval under both AAR and ERT. Results are shown for varying true treatment effect, and varying exacerbation rate in the control group

Figure 2 shows coverage for AAR and ERT definitions of at‐risk period, again with a recovery rate ratio of 1. Both methods show slight undercoverage compared to the nominal 95% level (means, 0.946 under AAR and 0.945 under ERT).

**Figure 3:**
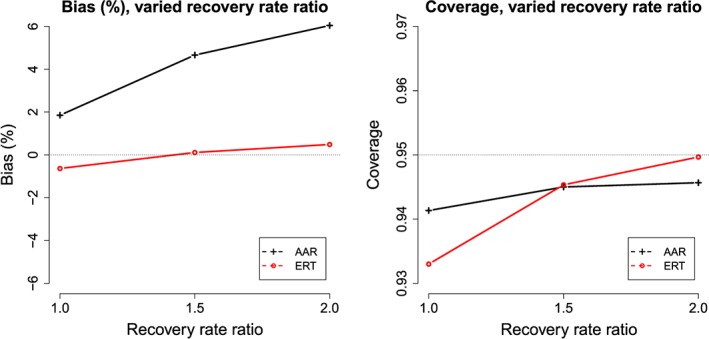
Percentage bias in treatment effect estimate and coverage probability of treatment effect confidence interval under both AAR and ERT. Results are shown for varying recovery rate ratio

Figure 3 shows how bias and coverage respectively are affected when there are differing recovery rates between the control and experimental arms as is possible if the drug reduced the duration of exacerbations. As the recovery rate ratio—the recovery rate on drug divided by the recovery rate on control—increases from 1.0 (no difference in recovery rates) to 2.0 (mean recovery time on drug is halved), the bias in the estimated exacerbation rate ratio increases by approximately 3.5 percentage points under AAR. The ERT negative binomial model remains unbiased throughout. Coverage is slightly poorer than nominal in both models when recovery rate ratio is 1, improving somewhat under both AAR and ERT as the recovery rate ratio increases.

#### 5.2.2 Power and type I error

How choice of at‐risk period affects the type I error rate and the power to show a statistically significant difference are shown in Table 4. Under ERT, the power increases by between 2% and 7% compared to AAR in the scenarios investigated; this is because the AAR definition of at‐risk period underestimates the overall rate of exacerbation and hence the rate ratio between the two treatment groups. The type I error probabilities were found to be 0.05 and 0.06 under the AAR and ERT approaches, respectively; both are close to the nominal 0.05. The slight inflation of type I error under ERT lessens as exacerbation rate on control decreases. Equivalently, power increases with exacerbation rate on control.

**Table 4:** Probability of rejecting the null hypothesis (power for non‐null treatment effects, Type I error for null case).

**Treatment Effect**	**Control Exacerbation Rate (per‐person year)**	**AAR**	**ERT**
0.5	1.3	0.82	0.83
	1.8	0.87	0.88
	2.3	0.89	0.91
0.7	1.3	0.34	0.35
	1.8	0.38	0.39
	2.3	0.39	0.42
1.0 (null case)	1.3	0.05	0.05
	1.8	0.05	0.06
	2.3	0.05	0.06
1.5	1.3	0.47	0.49
	1.8	0.51	0.54
	2.3	0.52	0.56

Abbreviations: AAR, always at risk; ERT, excluding recovery time.

## 6 SAMPLE SIZE SAVINGS

### 6.1 Formula

It is possible to calculate a required sample size correctly—that is, taking account of not‐at‐risk periods, even if those estimates assume AAR. Suppose we have a time‐based estimate of the baseline exacerbation rate under an AAR calculation for the control drug. Denote this estimated exacerbation rate for a patient on placebo as ${\hat{\mu }}_{\mathrm{AAR}}$. If the mean length of exacerbation was also recorded for each arm, then it is possible to calculate the corresponding estimate of the exacerbation rate under an ERT calculation, under a first order approximation, using the formula

(1) $${\hat{\mu }}_{\mathrm{ERT}}\approx \frac{{\hat{\mu }}_{\mathrm{AAR}}}{1-\frac{{\hat{\mu }}_{\mathrm{AAR}}}{\gamma }},$$

where *γ* is the recovery rate per person‐year on placebo and is the reciprocal of the expected duration of exacerbation in years. If we expect similar results in the upcoming trial, the required sample size can be calculated, taking account of periods spent not at risk, using the following sample size formula for the negative binomial distribution derived by Keene^11^:

(2) $$n={\left\{\frac{z_{1-\beta }+z_{1-\frac{\alpha }{2}}}{\log\left(\frac{\mu_{1}}{\mu_{2}}\right)}\right\}}^{2}\left\{\frac{\mu_{1}+\mu_{2}}{\mu_{1}\mu_{2}}+2\tau \right\},$$

where *μ*_1_ , *μ*_2_ represent exacerbation rates on treatment and control, respectively, calculated from Equation (1) and the assumed true treatment effect. Note that this is the sample size required per arm. This formula takes overdispersion into account, assuming the overdispersion is the same in each of the treatment groups. If there is no a priori estimate of overdispersion, one should consider choosing a conservative estimate.

### 6.2 Application to macrolide study

In the macrolide study, an exacerbation rate of 2.0 per person‐year on placebo under AAR was reported, when weighting patients by length of follow‐up. Assume that the true treatment effect size for our planned trial is equal to 0.5, i.e., the ratio $\frac{\mu_{2}}{\mu_{1}}=0.5.\;$Mean duration of exacerbation in placebo group was approximately 18.9 days, giving a recovery rate per person‐year of γ=365118.9=19.3. Converting this estimate of exacerbation rate to an estimate under ERT gives an exacerbation rate per episode‐free person‐year of

$${\hat{\mu }}_{1}=\frac{2.0}{1-\frac{2.0}{19.3}}$$

=2.2

for patients on placebo. This rate can now be used in Equation 2 in conjunction with the true treatment effect size and an estimate of the overdispersion parameter, *τ* *,* to calculate the required sample size. Our estimate of the overdispersion parameter was 0.98 (95% CI, 0.66‐1.87).

The required sample size using a baseline exacerbation rate calculated in the design under ERT compared to under AAR is shown in Table 5, where the analysis model uses the unbiased ERT model (columns 1 and 2). The sample sizes are shown for a series of true treatment effects ranging from 0.40 to 0.90. For example, with an estimated 2.0 exacerbations per person‐year on placebo calculated from previous data under an AAR model, to detect a statistically significant effect with 90% power assuming a true treatment effect of 0.7 at the 5% significance level we would require 526 patients (second column). However, if we realize that the exacerbation rate is an underestimate, taking into account recovery times, the exacerbation rate on placebo is approximately 2.2 per episode‐free patient‐year, and we would need only 504 patients (first column). The saving in sample size is modest and does not change markedly as treatment effect varies, remaining at approximately 5% throughout.

**Table 5:** Sample size required (ERT) and deemed to be required (AAR) for a trial with exacerbation rate on placebo of 2.0 exacerbations per person‐year (estimated under AAR), mean length of recovery of 18.9 days, overdispersion parameter 0.98, power 0.9 and type I error probability 0.05

**Baseline Rate**	**Calculated Using ERT**	**Calculated Using AAR**		**Calculated Using AAR**	
**Analysis model**	**ERT**	**ERT**		**AAR** a	
Treatment effect $\frac{\mu_{2}}{\mu_{1}}$	**N**	**N**	**Increase (%)¥**	**N**	**Increase (%)¥**
0.40	90	94	4 (4%)	102	12 (13%)
0.45	114	118	4 (4%)	132	18 (16%)
0.50	146	152	6 (4%)	174	28 (19%)
0.55	190	200	10 (5%)	232	42 (22%)
0.60	256	266	10 (4%)	322	66 (26%)
0.65	352	366	14 (4%)	460	108 (31%)
0.70	504	526	22 (4%)	698	194 (38%)
0.75	764	794	30 (4%)	—	—
0.80	1254	1304	50 (4%)	—	—
0.85	2336	2426	90 (4%)	—	—
0.90	5502	5710	208 (4%)	—	—

Parameter values on the basis of macrolide study above.

a Assuming treatment effect underestimated by 5% under an AAR model for treatment effects of 0.70 and below. **¥** percentage difference from column 1.

However, the true cost of using an AAR model for both the design and analysis is far more pronounced because the estimated treatment effect in this trial will be biased toward the null, and hence, the nominal power will be less than anticipated in the sample size calculation (see Table 5). In our simulation (Section 5) the relative bias was found to be approximately 5% in studies with a treatment effect of 0.7, with the bias increasing with higher treatment efficacy. Thus, assuming a bias of 5% under AAR models, to obtain 90% power the sample size for a trial designed and analyzed under an AAR model for an effect that has reduced toward the null from 0.7 to 0.735 would require 698 persons (Table 5, third column) and across the range of effect sizes from 0.4 to 0.7 between 13% and 38% more people are required than a trial designed and analyzed under an ERT model (Table 5, column 1). The relative bias in treatment effects between 0.7 and 1.0 is expected to be less than 5%, and therefore, we have not reported these scenarios. Nevertheless, there will still result in a loss of power under an AAR analysis for such scenarios.

## 7 DISCUSSION

The results show that to assume a COPD patient is always at risk of an exacerbation episode dilutes the estimated treatment effect between the drug and placebo groups. This effect is magnified as baseline exacerbation rates increase, as the treatment effect increases, and as recovery rate ratio increases. This can have important effects on the power of the trial and the type I error rate. Therefore, researchers should properly specify the at‐risk periods in which exacerbation onset can truly occur under their definitions of onset and recovery. This does not apply solely to the area of COPD exacerbations, but any situation in which events have a duration associated with them—in particular, when such durations may vary. Other examples of such data are hospitalization episodes for psychiatric disorders and system failures that require shutting down the system to make a repair.^13^

Exacerbations and their associated durations are heterogeneous events, and their definition with a simple mathematical formula has limitations. For example, a rule‐based definition might indicate that an exacerbation period consists of two or more overlapping events, but in reality the worsening of symptoms during recovery process was due to ending of acute therapy or that a particular treatment was not initiated at first presentation. However, the validity of the statistical methodology described in this paper is relevant no matter how exacerbations and associated durations are identified. Even when clinical trials have definitions in place, it may be wise to seek expert clinical adjudication of overlapping or tightly spaced exacerbations.

A possible limitation of the proposed approach is that there may be missing data in the reporting of episode durations. It is likely that the duration of some exacerbations will be missing because of patients not recording daily symptoms as patients may undergo emergency admittance to hospital or not wish to take study material on holiday. Our approach to missing data is to use single imputation of the mean exacerbation duration in each group. This does not account for the uncertainty in the missing data; an alternative approach would be to impute missing values using a multiple imputation procedure. Ideally, however, a new trial that uses the ERT approach should place equal importance on ascertaining both the beginning and ending of an exacerbation episode to address the problem of missing data.

In our simulation study we considered a COPD trial with exacerbation and recovery rates similar to other published trials.^4^, ^6^ Nevertheless, the scope of the simulations did not allow for between‐patient heterogeneity in recovery rates, nor did we address seasonal effects; these may be considered limitations of the simulation study that could be further investigated in future work.

To use the ERT approach in a clinical trial would require daily monitoring of patients to determine when they have recovered from their exacerbation. Electronic diaries are available and have already been used in some important clinical trials in COPD, such as, the FORWORD^20^ and SPARK^21^ studies for the purposes of exacerbation detection but were not used to monitor the duration of exacerbations. Electronic diaries are costly to implement involving purchase/hire, staff time for clinic visits to download data and train patients. Our study suggests that these costs would be offset against the reduced trial sample size required using the ERT approach. More work should be undertaken to determine the best diary questions, and their weightings, to determine the precise point that the patient has recovered from their exacerbation.

In summary, taking into account periods of episode recovery when designing trials or analyzing data containing episodic data is highly recommended. If this characteristic is ignored, then the estimated treatment effect may be underestimated.

## Supporting information

Supplementary Table 1: Power and Type I error under normal and missing not at random (MNAR) follow‐up times (using default parameter values).
Supplementary Table 2: Percentage bias under normal and missing not at random (MNAR) follow‐up times (using default parameter values, including treatment effect *ϕ* = 0.7).

Data S1 Supporting info item
