# Supplementary material for: Misspecification of at‐risk periods and distributional assumptions in estimating COPD exacerbation rates: The resultant bias in treatment effect estimation
Source: Pharm Stat. 2016 Dec 14;16(3):201–9. doi: 10.1002/pst.1798 (PMC5434805; doi:10.1002/pst.1798)
Supplement: Supplementary file 1 — Data S1 Supporting info item [file PST-16-201-s001.docx]

**Supplementary material: Misspecification of at-risk periods and distributional assumptions in estimating COPD exacerbation rates: the resultant bias in treatment effect estimation**

**Association between follow-up time and exacerbation rate**

In the comparisons detailed in the main body of the paper, the trial data are simulated under the assumption that the distribution of patient follow-up time follows a normal distribution with mean 365 and standard deviation σ=30. However, patients with higher rates of exacerbation are more likely to withdraw early [7]. To address this, another series of trials was simulated, taking this into account by associating exacerbation rate with length of follow-up in the following way:

With patient *i* exacerbating at rate *η_i_*,

*t_i_* ~ Exp(dη_i_),*

*T_i_ = min(t_i_*, 365),*

where *d* represents some fixed *drop-out* rate with range (0, 1], *t_i_** represents time until drop-out and *T_i_* represents time of drop out truncated to length of study, 365 days in this case. In this model, length of follow-up depends on exacerbation rate, and is greater for patients with lower exacerbation rates compared to those with higher exacerbation rates. Using the default parameter values noted in Table 3, we compare the effect of using ERT and negative binomial regression when follow-up time is related to exacerbation rate. Given the default exacerbation rate on placebo of 1.8exacerbations per year under ERT, drop-out rate was set to *d* = 0.6 to recover an approximate mean follow-up time of 1/*dη_i_*  = 1 year.

In comparison to previous scenarios, where follow-up time is unrelated to exacerbation rate, the power to show a statistically significant difference assuming a true difference of *φ = 0.7* (Supplementary Table 1) decreases by approximately a factor of 0.4 under both AAR and ERT, while bias increases (Supplementary Table 2)... However, the bias remains less extreme under ERT. Change in coverage was negligible under both AAR and ERT.

|  |  | **Power** | | **Type I error** | |
| --- | --- | --- | --- | --- | --- |
| Treatment effect (rate ratio) | |  | |  | |
|  |  | **Normal follow-up** | **MNAR follow-up** | **Normal follow-up** | **MNAR follow-up** |
| Negative binomial | AAR | 0.38 | 0.23 | 0.05 | 0.06 |
|  | ERT | 0.39 | 0.24 | 0.06 | 0.07 |

Supplementary Table 1: Power and Type I error under normal and missing not at random (MNAR) follow-up times (using default parameter values).

|  |  | **Bias** | | **Coverage** | |
| --- | --- | --- | --- | --- | --- |
|  |  | **Normal follow-up** | **MNAR follow-up** | **Normal follow-up** | **MNAR follow-up** |
| Negative binomial | AAR | 2.4% | 5.0% | 0.95 | 0.94 |
|  | ERT | 0.0% | 3.2% | 0.95 | 0.94 |

Supplementary Table 2: Percentage bias under normal and missing not at random (MNAR) follow-up times (using default parameter values, including treatment effect ).
